# Jobcenters’ strategies to promoting the inclusion of immigrant and native job seekers: a comparative analysis based on PASS survey data

**DOI:** 10.1186/s12651-022-00313-8

**Published:** 2022-07-11

**Authors:** René Lehwess-Litzmann, Janina Söhn

**Affiliations:** Soziologisches Forschungsinstitut Göttingen (SOFI) e.V., an der Georg-August-Universität, Friedländer Weg 31, 37085 Göttingen, Germany

**Keywords:** Immigration, Integration, Jobcenter, Public employment services, Active labor market policy, ALMP, Training, Labor market, Welfare state, H41, I38, J08, J15, J68, J61

## Abstract

This paper comparatively analyzes strategies of German Jobcenters to bring native and immigrant job seekers into employment. It focuses on clients who receive means-tested basic income for the unemployed, based on data from the Panel Study Labour Market and Social Security (PASS) from year 2015 to 2020. By way of logistic regression, the study identifies the impact of being an immigrant on the clients’ probability of reporting different kinds of offers like job referrals or courses, controlling for a number of other influential factors. The study also looks deeper into the effects of immigrant-specific attributes, such as heterogeneous German language skills. We found that the likelihood of offers by Jobcenters largely depends on the amount of time since immigration. Recent immigrants have the lowest chance of reporting most of the studied measures of active labor market policies. For immigrants having stayed more than 4 years in Germany, however, we do not find a disadvantage, and some measures out of Jobcenters’ toolbox are even more often offered to the longer-settled immigrants than to native clients. A possible explanation for the moderately under-average support of recent immigrants in terms of Jobcenters’ measures could be an institutional focus on improving German language skills prior to approaching the labor market.

**Supplementary Information:**

The online version contains supplementary material available at 10.1186/s12651-022-00313-8.

## Introduction: Jobcenters as crucial actors for the integration of immigrants?

Germany has witnessed successive waves of immigration since the World War II, in recent years especially from Eastern and Southern Europe in the context of the enlargement of the European Union (EU) as well as from Syria, Iraq, Afghanistan, and most recently Ukraine, due to wars and humanitarian crises. For successful integration of immigrants in German society, finding (decent) employment is one of the most important steps, maybe *the* most important one. Yet, immigrants face a higher risk of becoming and staying unemployed than natives (Integrationsbeauftragte 2019, p. 203; Kogan 2005). Furthermore, with the exception of recruited immigrants who enter Germany with an employment contract in hand, most immigrants do not have a job upon arrival (Söhn 2019, p. 51). They are significantly overrepresented among the unemployed in Germany, with consequences such as a high poverty risk (Bundesregierung 2021, pp. 50, 129, 210). Despite Germany’s booming economy, their relative share among job-seeking recipients of basic-income support for the unemployed (ALG-II [*Arbeitslosengeld II*]) increased between 2015 and 2020 (BA 2020 and older versions), i.e., our episode of investigation. Hence, public employment services (PES) is an institution of paramount importance for many immigrants’ labor market integration.

The PES do much more than granting unemployment benefits; they also monitor job offers and deploy a wide range of active labor market policy (ALMP) measures. For example, they can offer subsidized courses, which may, for financial reasons, be the only chance to access further education for some groups of clients, e.g., long-term unemployed individuals receiving means-tested benefits. Arguably, the PES are even more important for job-seeking immigrants than they are for native job seekers. This is due to immigrants’ lack of country-specific knowledge, networks, vocational certificates, and of other resources.

When individuals register for unemployment benefits, the main objective of both social law and PES is to help them become financially independent again by bringing them back into work, and this very often corresponds to the clients’ aim as well. Sometimes, however, the client, the PES, or both, regard immediate re-employment as unrealistic or even undesirable under the overall circumstances. Instead, some clients may require more knowledge of how to apply for jobs and present themselves in job interviews. Others want to or have to profoundly re-orientate their professional goals; this may be due to health issues or long episodes of unpaid care work in the family. Still, others consider attaining new qualification as a necessary intermediary step to reach the final goal of labor market integration or to gain employment on a higher level of qualification and income than previous competences and qualification would allow. In all those instances, the PES can offer help using their ALMP toolbox.^1^

This article investigates whether, and to what extent, PES in Germany treat immigrant and native clients differently with regard to efforts of placing them in regular employment and channeling them into various kinds of ALMP programs. Are there different patterns of what PES caseworkers offer to immigrated job seekers compared to German-born ones?

The percentage of non-German citizens among all participants of PES-subsidized further vocational training increased from 19.7 to 34.1 percent between 2015 and 2019, the average across this period being 28.4 percent; thus only one percentage point below the average share of immigrants in all job-seeking benefit recipients (29.3 percent) (BA 2019c, authors’ calculation). As to other activation measures, the share of foreigners varies little (BA 2019a, p. Tab 1.1). Older research based on quantitative micro data is inconclusive regarding immigrants’ access to PES-subsidized further training, showing a negative effect of foreign or non-EU citizenship, but a positive one of a short stay in Germany. A recent vignette survey among caseworkers in German Jobcenters, focused on refugee clients, showed that whether caseworkers recommended employment, occupational training or other programs as the first option depends on the socio-economic background and family situation of fictitious clients (Dietz and Osiander 2019). Boockmann and Scheu (2019, pp. 408, 412) find that Jobcenter staff consider an improvement of language skills and qualification of refugees as the prerequisite for finding qualified work, and thus for sustainably ending benefit receipt. However, such a long-term perspective is not systematically implemented, as both clients and case managers see also downsides of postponing contact with the labor market by lengthy “chains of subsidized measures” (ibid.). Many refugee clients urgently wish to start working, even in ‘bad’ jobs, and some case managers warn that receiving social benefits for several years might make clients lose sight of the aim of employment (ibid., pp. 412–413). Boockmann and Scheu (2019, pp. 412–415) recommend Jobcenters to combine education and training on the one hand with labor-market programs on the other (ibid., pp. 413 et seq.). According to qualitative findings by Schneider et al. (2008, p. 30), discriminating treatment by the PES seems to be a rare case, while Sauer (2010) reports such experience for some unemployed immigrants she interviewed, but again without comparison with native clients. Experimental correspondence tests, however, do show a lower quality of German municipalities’ and Jobcenters’ responses to email requests of ‘Turks’ versus ‘Germans’ (Grohs et al. 2016; Hemker and Rink 2017). Aggregate administrative statistics are descriptive only and hardly differentiate participants according to social or immigrant-specific characteristics (usually only gender, age, non-German citizenship or sometimes refugee status).

Finally, there are good reasons for investigating the foreign-born in a broad sense; while recent refugees have gained quite some attention in labor market research (Bonin et al. 2020; Boockmann and Scheu 2019; Dietz et al. 2018; Fendel 2019; Kasrin et al. 2021; Kosyakova 2020) in the context of the large influx of Syrian refugees in 2015/2017. In the long run, a comprehensive look on immigrants in general is called for. Quantitatively important groups like EU immigrants from Southern and Eastern Europe have and will continue to make up a substantial share of immigrants.^2^ Also, naturalized immigrants disappear in administrate statistics but deserve being included, for instance, as an immigrant group contrasting with recently arrived ones. Furthermore, as our interest lies on ALMP opportunities given by Jobcenters to their clients, rather than actual usage thereof (an outcome in its own right), we need to investigate offers received. Even though an offer does not equal its acceptance, receiving an offer is still the *sine qua non* for being allowed to participate in a program subsidized by the PES. Overall, to our knowledge, there are no representative and multivariate studies on immigrant-native differences or intragroup comparisons among immigrants regarding Jobcenters’ strategies for individual job-seeking clients.

Concretely, our analysis pursues the following research questions: *Which ALMP measures do Jobcenter staff offer to unemployed recipients of basic-income transfers, depending on whether they are natives, recently arrived and longer-settled immigrants? Does migration experience play a decisive role, once other influential factors (like age or family status) are considered? How are additional immigrant-specific factors like legal status, region of origin and German language skills linked to Jobcenters’ offers?*

Our contribution on German Jobcenters analyzes data from the Panel Study Labour Market and Social Security (PASS), a survey administered by the Institute for Employment Research (IAB) of the German Federal Employment Agency (BA). This data is nationally representative for basic-income recipients. Respondents registered as job seekers during at least one of the waves, from 2015 to 2020, answered the survey question whether the PES referred them to specific job openings regarding regular or marginal employment or offered them various ALMP measures.

Section 2 elaborates on the hypotheses based on conceptual considerations and previous research in our field. Section 3 presents the database, operationalization, and methods applied. After a descriptive analysis (Sect. 4.1), we will introduce Jobcenters’ offers as dependent variables in multivariate regression models (Sect. 4.2). In a first step, the study analyzes natives and immigrants together, in a second step, it looks at immigrants only, as this permits extending the model by additional, immigrant-specific variables. Critically reflecting on our database, we will discuss methodological challenges of the PASS-items on “vocational training and other courses” and present robustness tests (Sect. 4.3). The quantitative analysis is complemented by some insights from selected expert interviews with caseworkers and middle-management employees of Jobcenters, which were carried out in the context of a larger research project.^3^ The article finishes with a summary of the main findings and further discussions.

## Conceptual considerations and previous empirical insights

German law distinguishes jobless individuals who obtain unemployment insurance benefits based on prior contributions (Social Code [SGB] III) from jobless persons who receive means-tested basic-income support (SGB II). The latter group includes both (long-term) unemployed persons who have exhausted their entitlement to unemployment insurance benefits and job seekers who did not contribute to the insurance (long enough); among them recently arrived refugees who received political asylum or another form of humanitarian legal status.^4^ In the German two-tiered system, the so-called Jobcenters are the PES responsible for all job seekers without insurance benefits, thus for all who receive tax-funded welfare. Our empirical analysis is dedicated to Jobcenter clientele.

The priority for Jobcenters is to help clients end or reduce benefit receipt, as this is what social law requires (§ 1 SGB II). For most clients, this means that (non-subsidized) employment is the main goal, be it for the first time or after a period of joblessness. Benefit recipients generally are obliged to look for potential employers; Jobcenters may also refer clients to open positions and encourage them to apply. Jobcenters also pursue the aim of “maintaining, improving, or re-establishing the employability” of the client (ibid., authors’ translation). To this end, there is a range of ALMP measures which Jobcenters can implement (based on § 16 SGB II). In this respect, and in contrast to direct access to non-subsidized jobs, caseworkers in Jobcenters act as crucial gate keepers. In accordance with the administration’s general goal of economical and effective spending, offering an ALMP program is a discretionary decision which caseworkers as “street-level bureaucrats” (Lipsky 2010) can opt for. They consider the degree of their clients’ employability, the likelihood of successfully finishing an educational program, with respect to the clients’ motivation and learning ability, as well the probability of the newly acquired knowledge helping to find an appropriate job thereafter (Yankov 2010, pp. 8, 24–25). Having to respect their organizations’ budget restrictions, caseworkers try to identify those clients for whom a program seems most promising. Vocational (re-)training programs and long-term subsidized employment schemes are among the most substantial and costly offers Jobcenters can make. Cheaper short-time programs do not involve potential lock-in effects (standing in the way of immediate transition into employment), which are typical for longer programs (see the evaluation of ALMP programs' effects on refugee clients by Kasrin et al. 2021, p. 3).^5^

Clients themselves also have to consider pros and cons of looking for (non-subsidized) work versus attending (longer) programs while receiving the rather meager basic-income support. Course attendance and job search do not utterly exclude one another, but each restricts time and mental energy that remain for the other option. Individuals more or less consciously weigh possible future occupational advantages of the qualification, knowledge, or skills to be gained against their self-perceived learning capacity and motivation, individual or family-related obstacles or resources as well the opportunity costs of not having income from working (Becker 2019, pp. 3–5; Cross 1981, pp. 98, 116–117). An experimental study embedded in a survey of refugees showed that given the hypothetical scenario of having been offered a low paid job and vocational training, respondents were more likely to choose the latter; the higher the future earnings after the specific vocational training, the easier the exam was said to be, and the higher the apprenticeship wage during training (Damelang and Kosyakova 2021, pp. 6–8). Again with regard to refugees, Boockmann and Scheu (2019, p. 409) highlight the high motivation of many clients to succeed in Germany, but also the potential demotivation by the lengthy process of overcoming various administrative and qualification-related hurdles standing in the way of job uptake. Based on interviews with Jobcenter staff, they find that the wish for economic independence from Jobcenters can “reduce the readiness to go through qualification measures which would allow them enter higher segments of the labor market in the long term” (ibid, p. 409, authors’ translation).

Hence, offering and accepting ALMP programs are no isolated decisions; it rather takes place in a context where employment is the major competing alternative. Clients may communicate their preferences to their caseworker and possibly influence the latter’s decision. On the one hand, given the asymmetric power relation, an offer by the Jobcenter is a necessary condition of the clients’ participation in an ALMP program.^6^ On the other hand, substantial measures like 2-year vocational re-training or professional coaching are too expensive to force clients to attend. They tend to be a privilege that clients have to fight for and be knowledgeable and confident enough to do so (regarding immigrants see Schneider et al. 2008, p. 18).

In some cases, clients’ rights limit caseworkers’ discretion over ALMP measures. Participating in “integration courses”, regulated in the Residence Act (*Aufenthaltsgesetz*, §§ 43 ff.), is both a right and an obligation for certain groups of, primarily, newly arrived immigrants.^7^ These courses mainly consist of German language classes plus civic education. EU nationals may participate, too, but are neither obliged to nor have the right to do so.

Overall, the question whether Jobcenters make different offers to immigrant and native basic-income recipients comes down to caseworkers’ perceptions (Maynard-Moody and Musheno 2003) of these two groups with regard to the necessity, needs, motivations, likelihood of success, and maybe also personal ‘worthiness’, to be placed into regular employment or into some kind of ALMP measure. In the following, we will develop some hypotheses based on both theoretical arguments and related previous research.

### Referral to open job positions

First, this study looks at the goal of direct employment take-up. As for clients’ preferences, in terms of a disincentive of looking for work, immigrants might consider the high amount of German welfare benefits compared with salaries in their home country in absolute terms. However, living expenses in Germany is also higher than before migration. Immigrants might actually be more eager than natives to work immediately, as many of them feel obliged to send money to relatives back in their country of origin (on remittances see Sinning 2011). In addition, residence law makes their right to stay in Germany beyond their fixed-term residence permit depend on continuous pension insurance contributions (Knuth 2021, p. 56). In our expert interviews with Job Center staff, one employee put it this way:

*“This happens quite often, when it comes to working, the [migrant] clients say ‘I want work, no matter what kind, I will do anything’ … also the academically trained ones.” (Interview I)*

If Jobcenters shared this priority, as a theoretical assumption not tested by our data, *they could refer immigrants to open positions more often than natives (Hypothesis [H]* #*1a).* In addition, as these newcomers are less familiar with job search on the German labor market than natives, caseworkers might not want to rely on immigrant clients’ own initiative to find employment, but explicitly refer them to open positions*.*

In contrast to this potential scenario, Jobcenters (and clients) could be aware of immigrants’ high hurdles to find employment and decide that these should be tackled before making efforts of placing clients into employment. After all, many immigrants (and in particular recently-arrived ones) struggle with devaluation of their foreign qualification and/or work experience (Boockmann and Scheu 2019, p. 407; Damelang et al. 2020; Nohl et al. 2014, p. 24ff.), insufficient knowledge of formal and informal norms in the process of job search in Germany, actual or assumed German language deficits (Esser 2006, p. 400ff.), and ethnic discrimination in the German labor market (e.g., Veit and Thijsen 2019), resulting in both above-average unemployment rates, as discussed above, and below-average work quality (Gundert et al. 2020). Caseworkers may refrain from making clients apply in the first place when success is improbable. If caseworkers judged immigrants less employable than native job seekers, *they should refer immigrant clients to open job positions in regular employment less often than native (H #1b).*

Jobcenters may also refer unemployed clients to marginal employment (“*Mini-Jobs*” in German), i.e., employment with low earnings (up 450 Euros per month), low working hours, limited social insurance, and slim chances to lead to regular employment (Freier and Steiner 2008). Similar to ‘1-€-jobs’ (*Arbeitsgelegenheiten*) (Kasrin et al. 2021, p. 3), Jobcenters might consider marginal employment as an option mainly reserved for ‘difficult’ long-term unemployed clients. The latter are often faced with multiple employability obstacles so that Jobcenters regard regular employment as out of reach. Limited personal availability for work due to child care, for instance, can be an alternative reason for marginal employment. All these obstacles can apply to both native and immigrant Jobcenter clients. However, given the above-mentioned immigrant-specific obstacles to employment and also the particular need to bring refugee clients into contact with the labor market, *Jobcenters might suggest applying for marginal employment more often to immigrant clients (H #2).*

### Support for the job-search process

Getting insight and support on how to write applications and how to present oneself in a job interview should be particularly relevant for newly arrived immigrants as there are most likely small or large cultural differences (depending on the country of origin) in what is regarded as an appropriate form.^8^ Hence, we expect *unemployed immigrants to be offered support with applications and job search more often than their native peers (H #3)* (For our counter hypothesis regarding any type of ALMP program, see the end of this section).

Looking for a job can involve monetary expenses, especially travel costs to go to a job interview. Jobcenters routinely *reimburse application or travel costs* upon job seekers’ request. Thus, such an offer by the Jobcenter hinges on the prior decision by an employer to put the person on a shortlist and indicates a high level of employability. Given the obstacles immigrant job seekers face on the labor market, we expect *reimbursement of application or travel costs to be offered less often to immigrant than to native Jobcenter clients (H #4).*

### PES-subsidized (self-) employment

Some ALMP measures support employment more directly than the programs mentioned so far. They ‘create’ work experience and hence a potential bond with an employer. In the case of a so-called “measure with an employer” (*Maßnahme bei einem Arbeitgeber*), job seekers work for an employer during up to 6 weeks (in exceptional cases up to 12 weeks), who receives financial subsidies by the PES during that period (Arbeitsagentur 2019) and ideally hires the job seeker once the program runs out. According to Kasrin et al. (2021, p. 3), participating companies prefer job seekers with a comparatively high level of employability (Dietz et al. 2018). Indeed, such programs bring unemployed (male) refugees most quickly into regular non-subsidized employment compared with other AMLP measures (Kasrin et al. 2021, pp. 5, with further training as the second-best option). With respect to the immigrant-specific need to gain employers’ trust and also regarding recent immigrants’ eagerness to work, *the latter might be offered a program with an employer or an internship more often than native job seekers (H #5)*, provided the client is seen as sufficiently qualified.

Financial support to become self-employed could, on the one hand, be an option for immigrants as they have greater difficulties to find employment (Integrationsbeauftragte 2019, p. 203; Kogan 2005); on the other hand, successful foundation of one’s own business requires knowledge about administrative procedures and markets that *recent* immigrants tend not to have. Jobcenters offer self-employment support too rarely to statistically test a respective hypothesis with our data.

### Counselling on occupational goals and qualification pathways

Many immigrants are in need of (re-)orientation regarding their occupational future when faced with devaluation of their previous qualification and/or work experience (Damelang et al. 2020; Nohl et al. 2014), or when they have never worked beyond their household or the informal sector in their country of origin (Bonin et al. 2020, p. 73, regarding refugees). Courses which inform participants about different occupational fields in Germany or individual coaching could, as a first step, support such individuals in deciding whether to apply for jobs, apprenticeships, internships, vocational re-training or courses leading to full recognition of their foreign qualification. If such needs were paramount for the Jobcenters’ decision*, they should offer courses on occupational orientation or respective vouchers more often to immigrant clients than to native ones.* (H #6).

### Further training

Some immigrants have special needs for further vocational training. First, insufficient educational infrastructure, war, or discrimination in the country of origin could have prevented them from realizing their educational goals there. Second, insufficient transferability of immigrants’ foreign qualification can serve as a push factor to gain new or complementary qualification in the immigration country in order to prevent long-term social downward mobility (for Canada, the USA, Germany, and the Netherlands repectively see Adamuti-Trache 2011, pp. 75–76; Hashmi Khan 1997, p. 287; van Tubergen and De Werfhorst 2007, p. 885). In addition, caseworkers could be aware of the fact and communicate it to their clients that attending educational programs is indeed an effective, though strenuous and time-consuming, strategy to raise long-term chances of finding qualified work (Deeke and Baas 2013, regarding immigrants’ employment after further training financed by German PES; Kanas and van Tubergen 2009; Lancee and Bol 2017). Jobcenter-subsidized further training is even more successful in raising formerly unemployed persons’ incomes than subsidized employer-programs, while both participant groups fare far better than job seekers receiving no measure (Kasrin et al. 2021, p. 7, regarding refugees). These arguments feed into our hypothesis that if immigrants’ special need for further vocational training and the effectiveness of such programs were decisive—again, a theoretical assumption grounded in previous insights but not tested here—*Jobcenters should offer it to immigrants more often than to natives (H #7a)*. The opposite expectation (*H #7b*) is favored by the *time factor of attending a longer program* like a 2-year vocational re-training course, which might turn this option prohibitive for immigrants who already spent numerous months in German-language classes and now urgently want to earn their living rather than continue learning with only the modest welfare benefits available. Refugees’ case managers at times fear adverse effects of too-long (chains of) measures, possibly leading immigrants to become used to welfare transfers as a durable substitute for income from employment (Boockmann and Scheu 2019, p. 412).

The arguments put forth so far theoretically indicate a higher need of (recent) immigrants for ALMP programs than there is among native job seekers, and such needs could turn into actual choices on part of Jobcenters. However, there are two general reasons to expect *fewer* offers to immigrants: Jobcenters may, rightfully or not, consider immigrants’ linguistic ability too low to successfully follow a mainstream ALMP program in German. Furthermore, immigrants could be less knowledgeable, confident, or vocal about their preferences in favor of a suitable ALMP program in their communicative interaction with caseworkers (Holzinger 2020). Schneider et al. (2008, p. 30) report “situations in which immigrants feel disadvantaged and sense that their qualifications, competencies and career plans cannot be met” by the PES (Sauer 2010, p. 157). Relatedly, field experiments revealed some discrimination of German municipal governments (Grohs et al. 2016) and Jobcenters (Hemker and Rink 2017) regarding the response quality in reacting to email-requests from persons with typical Turkish (or Rumanian) names. Against this backdrop, *the counter-hypothesis of fewer offers to immigrants could hold for some or all of the above-mentioned ALMP measures*.

### Immigrant-specific influences

Taking a deeper look at migration-specific factors, we presume a *shorter duration of stay in Germany to lead to a larger difference in the treatment by Jobcenters compared with that of native clients (H #8)*. It remains to be seen whether this assumption holds once the level of German language skills is considered. *Good German skills would make language classes superfluous and should make both referrals to (regular and subsidized) employment and to ALMP courses held in German more likely (H #9).* As far as *legal status* and *nationality* are concerned, Jobcenters’ referrals to job vacancies (and reimbursement of travel cost) could be indirectly influenced by employers’ possible recruiting preference for white, non-Muslim and/or European immigrants (Koopmans et al. 2019; Veit and Thijsen 2019), as well as for those with German citizenship (Steinhardt 2011) or permanent residence status *(H #10)*.

The potential effects of migration status, hypothesized in this section, could be mediated by other personal and structural factors known to influence Jobcenters’ decisions more generally. Such potential composition effects could be based on, e.g., immigrants being younger on average than native job seekers, with higher age being negatively associated with participation in further training (Osiander 2019, p. 70). Furthermore, higher levels of education are a component of positive (self-)selection into employment and (further) education (Kruppe 2009, pp. 11, 14; Osiander 2019, p. 76), also among refugee clients (Dietz and Osiander 2019, p. table 3). Dependent children in one’s household are a possible hurdle as well (Kruppe 2009, p. 14). Finally, there are good reasons for investigating the foreign-born in a broad sense: while recent refugees have gained quite some scientific attention (e.g., Bonin et al. 2020; Boockmann and Scheu 2019; Dietz et al. 2018; Fendel 2019; Kasrin et al. 2021; Kosyakova 2020) following the large influx of Syrian refugees in 2015/2017, a comprehensive look on immigrants in general is called for. Quantitatively important groups like EU immigrants from Southern and Eastern Europe have and will continue to make up a substantial share of immigrants.^9^ Also, naturalized immigrants disappear in administrate statistics but deserve being included, e.g., as an immigrant group contrasting with recently arrived ones. The following sections shows our empirical study is hence adequately inclusive.

## Methods: database, operationalization, strategy of analysis

### Data and sample

The study uses the Panel Study Labour Market and Social Security (PASS) (Berg et al. 2020) as it contains a question on having received an offer of various ALMP programs by the PES (see below for details). In addition, the PASS (rather than administrative data) is well suited for this research as it contains important information necessary to study immigrant integration, namely the year of immigration for any foreign born, which allows to calculate the duration of stay as well as to identify naturalized immigrants.

We primarily use the first of the two samples the PASS consists of, i.e., the one representing households which receive basic-income support (the other one is sampled on the German population without further restrictions) (Hohmeyer and Wolff 2015, p. 13). Our investigation is limited to the year 2015 to 2020, as the item which is most crucial for our analysis is only included in the survey as of wave 9. Our target population consists of adults, aged 18 to 64, who were registered at a Jobcenter among those looking for work and receiving basic-income support. The sample was restricted to survey participants registered as job seekers, as only they are asked about Jobcenters’ offers during the last year. Furthermore, we excluded secondary-school students as well as recipients who were not in regular contact with the Jobcenter: Such contact is a precondition of a potential ALMP offer. However, this restrictions disproportionally excludes female refugees (Bähr et al. 2017).

In our unbalanced panel, a minority of participants of the PASS survey fulfilled our sample criteria for more than one wave in our observation period. We choose person-waves (instead of persons) as our unit of observation because each year that a person fulfills the criteria of receiving basic income and looking for a job, there is a new opportunity for offers to be made by Jobcenters to the client. We thus have a hierarchical data structure, with 8275 person-waves nested in 4954 interviewees. 61.8 percent of the observed persons appear in our sample only once (Table 5).

We define immigrants as foreign-born having arrived in Germany as adults.^10^ According to this definition, 45.3 percent of persons (and 39.1 percent of person-waves) in the observed population are immigrants. We further distinguish immigrants by their duration of stay, i.e., whether they have arrived in Germany up to 4 years ago or at least 5 years ago. Note that immigrants tend to be observed for a smaller number of survey waves than natives: 69.8 percent of immigrants and only 55.2 percent of natives are part of our sample during 1 year only.

### Items for target variables

For operationalizing Jobcenters’ offers, we draw on the survey item “Since your household has obtained Unemployment Benefit II [since our last interview, respectively], have you ever been offered the following by the Jobcenter?” (PTK1701). There is a battery of items to choose from, multiple answers possible. In our analysis, we consider the following offers^11^ (IAB’s translation of the survey items into English) and we connect them with the hypotheses in Sect. 2:

- *A part-time or full-time job or an apprenticeship*: Jobcenters refer clients to open job positions in the form of regular employment, subject to social insurance contributions, and encourage them to apply. Apprenticeships refer to the German dual vocational training in which learning takes place in a company and in a vocational school alternately. Apprenticeships are also subject to social security contributions. While the previous section formulated H #1a/b only comprises regular employment, its arguments also apply to apprenticeships in firms as far as caseworkers may view clients as (not) well-enough prepared to compete with other applicants.
- *Minor employment, e.g., a mini-job* (H #2)^12^
- *Support for your applications, e.g., assistance with the preparation and compilation of application documents* (H #3). This item partly overlaps with the one on vouchers, see below, as it can be implemented by private providers rather than by the PES.
- *Reimbursement of application costs or travel expenses:* though this is something Jobcenters can offer (H #4), it actually mirrors the “success” of having been invited to a job interview.
- *A program with an employer or internship* (H #5)
- *An activation or placement voucher with which you can choose a program yourself*: Financed by the PES, these vouchers give access to services by licensed private local providers of various kinds of programs. This item is not optimal for operationalizing our hypotheses as it refers to the *way* Jobcenter offer programs, rather than their content. Support with applications, occupational orientation, or further vocational training, of which only two are response categories of their own, can be granted via vouchers. The respective hypotheses (H #3, #6, #7) connected to these measures also apply to vouchers. Jobcenter statistics tell us that 56 percent of vouchers pertain to the policy goal of giving clients orientation about employment and vocational training (*Heranführen an Ausbildung und Arbeitsmarkt*) and more than two thirds of the vouchers actually redeemed do so (BA 2019a, table 5, authors’ calculation). On the PES website, courses which the vouchers are meant for refer to, e.g. assistance in job applications, self-marketing strategies for academically trained job seekers, immigrant-specific courses supporting economic integration, one-on-one application coaching, or business English. (H #6-plus)
- *Vocational training, retraining or a course*: This offer includes both courses lasting a few months and full vocational re-training taking 2 years. (H #7)
- *An integration course or another German course*: This item should exclusively apply to immigrant Jobcenter clients. It has been included in the survey only as of wave 10 (year 2016), so the case numbers are smaller in our models which include this item. German courses other than those which are part of the integration course are, e.g., classes for occupation-specific German targeting more advanced language learners. We study this target variable only with regard to the intra-group comparison among immigrant clients.

Caseworkers may offer several measures (or nothing at all) during one counselling appointment or in consecutive ones. The survey, however, asks respondents whether the Jobcenter has offered them any kind of offers since they became a job seeker or since the last time they took part in the yearly survey, respectively. During that time, more than one measure of the same type could have been offered and the sequence of those offers remains unknown. Hence, we measure whether a type of program was offered at least once during the reference period according to the respondents’ memory and willingness to answer correctly.

Another source of potential bias is that the system of labor market services also has a “memory”: a specific offer may be made only once (in consequence, persons observed for a smaller number of years could tend to have a higher chance of receiving an offer in a given year of observation). Yet, offers could also become *more* probable the longer the client is in the situation of receiving benefits and looking for a job. As a test of robustness (4.3), we will do an extra analysis where persons are considered only once across the whole observation period.

Our findings on shorter- vs. longer-settled immigrants could be biased in so far as some of the immigrants who arrived earlier could have left the country in the meantime, leading to some kind of social selectivity of the remaining longer-settled immigrants. Yet, this methodological problem cannot be overcome with available data.

Regarding one item, it is necessary to consider that questions are asked in a certain order in the context of the CATI/CAPI interviews (Jesske and Schulz 2018, p. 68), in which interviewers usually read out loud battery items one after the other. In case of items that overlap to a certain degree, the ordering can be consequential. It is thus unfortunate that the item *Vocational training, retraining or a course* is proposed to respondents before that of *Integration course or another German course*: it is likely that some of the immigrants who had been offered a language course in the reference year reported “yes” already when they read or heard of the item on vocational (re-)training and the undefined “course” mentioned here. Indeed, out of the 818 immigrants who reported the offer of *Vocational training, retraining or a course* in waves 10 to 13, 648 also reported to have been offered an integration/German course. There is no way to find out in retrospect how many of those with two positive answers have actually been offered *both* kinds of courses, and how many were *only* offered an integration or language course. However, chapter 4.3 presents a robustness test controlling for the described issue and further critical arguments.

### Predictor and control variables

With regard to our hypotheses on immigrant-specific features, the *duration of stay* in Germany (the time between immigration and the survey interview) is adequate to test H #8 and *level of linguistic competence in German* (respondents’ subjective evaluation)^13^ regarding H #9. Both *legal status* (temporary residence permit being the status of recognized asylum seekers and most recently arrived non-EU citizens)^14^ and *world regions of origin* (broad categories considering the number of observations) operationalize H #10. Note, however, that legal status and duration of stay do not provide any information on the time when German authorities grant the status of a legally recognized refugee and thus a (fixed-term) residence permit.

Several other individual and household-related characteristics of the observed population (including natives) serve as control variables in our multivariate analyses, including age, level of education, duration of the current unemployment episode, subjective health, and partner household. In addition, we assume gender to become relevant (only) when viewed in its interaction with own children to care for, also depending on children’s age (Leber and Möller 2008, p. 418). The under-employment quota (individuals registered as unemployed plus those enrolled in PES programs; BA 2019b) of the respective regional state controls for local employment opportunities.

Some predictor variables are inherently related to each other, e.g. duration of stay and respondents’ age (Pearson’s rho = 0.53): given our definition of immigrants as having arrived as adults, those with longer duration of stay cannot be very young adults at the time of the interview. We use only broad categories of years since arrival in order to avoid empty cells and hence attenuate the challenge of collinearity. Duration of stay is also linked to region of origin due to successive migration waves from different regions (e.g., most persons from the former USSR arrived in Germany between 20 and 5 years before the survey interview). Due to naturalization being granted after several years of residence and due to more generous rules applying to German resettlers, regions of origin are also strongly correlated with legal status (Cramér’s V = 0.58).^15^ All other independent variables’ correlations are inconspicuous.

### Methods

Seeking to analyze several different but not mutually exclusive outcomes, we estimate separate binary models for each dependent variable. The main interest of our analysis is on differences in “treatment” by Jobcenters between groups of persons, that is natives and immigrants. Individuals’ membership in these groups does not change over time in our sample (or only rarely, regarding the categorized duration of stay). As the majority of persons does not stay in the sample for more than one survey wave, the variance in the data which drives our results is derived from the differences *between* person-waves, rather than within persons across waves. Therefore, we choose simple logistic regression analysis (a robustness check with logistic random-effects models yield very similar results, cp. Sect. 4.3). By applying clustered standard errors at the person level, our models take account of the non-independence of repeated observations of the same person, where applicable.^16^ Results are presented as average marginal effects (AME), which can be read as the predicted average difference in percentage points regarding the probability of the dependent variable for a one-unit change of an independent variable, with all other predictors held constant.

While the multivariate analysis is based on unweighted data, descriptive results are always weighted (pertaining to person-waves). Weighting the data evens out disproportional numbers in the sample, e.g., persons form Syria and Iraq, who were oversampled in wave 10 (year 2017) of the PASS survey (Jesske et al. 2019, preface). The weighting parameters included in the PASS always refer to one specific survey wave. In order to determine values for the whole period of observation, we first calculate weighted values for each of the five panel waves separately and then average those values across all waves.

As a small qualitative add-on, we insert few selected excerpts of expert interviews we conducted during the larger mixed-methods research projects into which the analyses presented here was embedded. The interviews, anonymized in this article, with mid-level managers of Jobcenters and Employment Agencies in a mid-sized West-German city and in a more rural community took place in 2016/2017.

## Results and discussion

### Description of the reference population and Jobcenters’ ALMP offers

The first part of this section describes our reference population by some personal and household features of native and immigrant Jobcenter clients (Appendix Tables 6 and 7) as well as offers made to them by Jobcenters between 2015 and 2020 (Table 1). The shares reported always refer to the weighted yearly average across the 6 years studied.

**Table 1:** Jobcenters’ offers to job-seeking basic-income recipients: natives and immigrants, by duration of stay in Germany

	All job-seekers	Thereof…			
		Natives	Migrants	Thereof …	
				Up to 4 years of stay	At least 5 years of stay
Regular employment	31.8	31.8	31.7	24.5	38.1
Marginal employment	17.0	17.2	17.2	13.7	20.4
Assistance in applications	28.3	28.0	29.3	29.9	29.2
Reimbursement of application costs or travel expenses	43.0	47.6	31.9	29.0	32.5
Program with employer or internship	12.1	11.8	13.0	16.3	10.2
Fin. support to become self-employed	2.3	2.3	2.2	1.6	3.0
Activation or placement voucher	14.9	15.4	13.8	10.4	18.1
Vocational (re-)training or a course	19.6	14.2	31.9	39.8	26.8
Other offers	1.8	2.0	1.3	0.9	2.0
No offer, excl. integration or language course	29.5	29.0	31.4	31.2	30.4
Integration or language course	14.7	0.9	46.2	69.6	23.8
No offer, incl. integration or language course	26.6	29.0	22.3	15.8	27.9

Source: PASS waves 9–14, own calculations. Weighted mean yearly shares of clients who received at least one of the respective offers in the period 2015–2020

Compared with the natives in our observed population, the share of men is higher among immigrants (59.8 vs. 52.5 percent), in particular among those immigrants having arrived during the recent 4 years (73.4 percent men). Immigrants without any professional qualification are clearly overrepresented (54.9 vs. 39.4 percent among natives), with 61.2 percent among the recent immigrants. Yet, the share with an academic qualification is also considerably higher among immigrants studied here (16.8 vs. 3.6 percent among natives). In the native group, a majority (57.0 percent) has a degree of non-academic vocational training (vs. 28.3 percent of immigrants). The foreign-born Jobcenter-clients live far more often with a partner (61.7 vs. 28.8 percent) and with children than their native peers.

Among the immigrants, 13.2 percent are German citizens and 19.4 percent citizens of another EU country (Appendix Table 7). Among third country nationals, the share with a temporary residence permit is higher than with a permanent one. This holds in particular for immigrants living in Germany for less than 5 years (65.0 percent with temporary residence permit). Among those who give valid answers to the respective survey question, a high share of the recent immigrants say they came to Germany as asylum-seekers: 86.4 percent in 2018 (wave 12) and 69.3 percent in 2019 (wave 13). Persons from the former Soviet Union and Eastern Europe constitute the largest shares of non-recent immigrants (30.8 and 26.0 percent, respectively). The majority of those having stayed in Germany for less than 5 years stem from (Western) Asian countries (62.5 percent), including countries many refugees fled from.^17^ Looking at immigrants’ German language skills, 36.4 percent among recent immigrants and 54.4 percent among the longer-settled ones consider their own skills as good or very good.

Regarding the different measures which the PES can offer to job-seeking clients, Table 1 shows no major difference between the proportions of native and (all) immigrant clients referred to job vacancies pertaining to regular employment (31.8 vs. 31.7 percent). Yet, distinguishing immigrants by duration of residence reveals a substantial gap. In accordance with some of our expectations, fewer recent immigrants (24.5 percent) report such referrals, but 38.1 percent of the longer-settled immigrants do. The latter also received referrals to marginal employment more often (20.4 percent) than natives (17.2 percent) or recent immigrants (13.7 percent). By contrast, Jobcenters offered support with job applications most often to recent immigrants (29.9 percent). Natives feature the highest share of those offered reimbursement of application costs and travel expenses (47.6 vs. 29.0 and 32.5 percent among recent and longer-settled immigrants respectively). Probably, native Jobcenter clients are invited more often to job interviews than immigrant clients.

With the exception of assistance in applying for jobs, the measures reviewed so far depend strongly on labor market conditions and employers’ decisions. In contrast, caseworkers’ own considerations are more decisive for offers regarding the other elements of the ALMP toolbox. Jobcenters offered a subsidized program with an employer or an internship most often to recent immigrants (16.3 percent). Financial support to become self-employed is rarely offered (2.3 percent of the observed population, and most often non-recent immigrants, 3.0 percent). Activation or placement vouchers, often meant for personal occupational orientation but also for further training or support in the job search, show a similar pattern as referrals to (regular or minor) employment. Recent immigrants receive them least often (10.4 percent) and longer settled immigrants more often (18.1 percent), with native clients in between (15.4 percent).

Concerning occupational (re-)training “or other courses”, by contrast, recently arrived immigrants report such offers most often (39.8 percent), natives least often (14.2 percent), the other immigrants positioned in between (26.8 percent). As mentioned above, however, we cannot be sure that all respondents kept this response category “clean” of integration and language classes (see Sect. 4.3 below). The integration courses are “offered” to 69.6 percent of recent immigrants, they are obligatory in many cases. Among those staying in Germany already more than 4 years, 23.8 percent reported the offer to attend integration and language classes.

Leaving aside such measures not applicable to natives, we find that 29.0 percent of native and 31.4 percent of immigrant clients (all in regular contact with their Jobcenter) do not receive any offer in an average year of observation. Once we include integration and language classes, the share of recent immigrants without offer from the Jobcenter is only 15.8 percent.

### Determinants of Jobcenters’ offers: multivariate analyses

The presentation of multivariate results has two parts: In the first, we analyze for the whole sample, natives and immigrants, in how far personal and context factors explain the clients’ chance of the Jobcenters offering them a specific type of employment policy measure and whether being an immigrant plays a significant role. In the second part, we run our model for immigrants only and add immigrant-specific variables in order to differentiate this heterogeneous group. This helps us to look deeper into the conditions under which Jobcenters make offers to immigrants.

#### Natives and immigrants compared

The joint model for immigrants and natives in Table 2 confirms most of the above descriptive findings (cp. Table 1). It is again paramount to distinguish immigrants with shorter and longer duration of stay because effects sometimes point in opposite directions. For those immigrants who have arrived up to 4 years before the interview, the estimated probability to be referred to a *regular job or an apprenticeship* is on average 12.3 percentage points (p.p.) lower than that of native clients, while there is no significant difference between natives and longer-settled immigrants. H #1a—the expectation that the PES offer more jobs to immigrant clients in general—is thus not supported by our findings, while there is evidence which corroborates H #1b. Possibly, Jobcenter staffs do not consider recent immigrants yet fit for employment or they anticipate that it is harder for them to meet employers’ requirements (e.g., German language skills). With regard to *minor employment*, caseworkers are most likely to refer longer-settled immigrants to job vacancies (6.0 p.p. more than native clients), with no difference between natives and recently arrived immigrants. H #2 is thus supported only for part of the immigrant clientele.

**Table 2:** Determinants of various ALMP measures by Jobcenters to job-seeking recipients of basic-income support (joint model)

Independent variables	Dependent variable: referral/offer made by the Jobcenter							
	Regular employment	Marginal employment	Assistance in applications	Reimbursement of application or travel costs	Program with employer or internship	Activation or placement voucher	Vocational (re-)training or course	No offer^a^
Person category (reference: native basic income recipients looking for a job)								
Basic income recipients looking for a job who immigrated during the past 4 years	− 0.123***	− 0.007	− 0.053***	− 0.201***	0.015	− 0.069***	0.139***	0.101***
Basic income recipients looking for a job who immigrated at least 5 years ago	0.039	0.060***	0.057**	− 0.114***	0.012	− 0.013	0.115***	0.008
Age (reference: 35 to 44 years)								
18 to 24 years	0.035	0.053*	0.103***	0.048	0.131***	0.009	0.005	− 0.075***
25 to 34 years	0.015	0.024	0.032	0.046*	0.030*	0.011	0.014	− 0.035*
45 to 54 years	− 0.032	0.003	− 0.050**	− 0.056**	− 0.015	− 0.035**	− 0.054***	0.070***
55 to 64 years	− 0.089***	0.005	− 0.089***	− 0.090***	− 0.038**	− 0.063***	− 0.126***	0.142***
State of health: bad (reference: very good to less good)	− 0.028	− 0.008	− 0.035	− 0.069***	− 0.025	− 0.034*	− 0.056**	0.043*
Professional qualification (reference: none, lower-secondary school-leaving certificate at most)								
None, but upper or intermediate secondary school-leaving certificate	0.031	− 0.033*	0.003	0.012	0.000	0.025	0.048**	− 0.002
Non-academic professional qualification	0.056***	− 0.018	0.031*	0.068***	0.002	0.032**	0.021	− 0.031*
Academic qualification (university or technical/teacher training college)	0.049*	− 0.070***	0.084***	0.101***	0.008	0.074***	0.046**	− 0.064***
Duration of current unemployment so far (reference: 12 to 23 months)								
0 to 2 months	− 0.063***	− 0.019	− 0.057***	− 0.093***	− 0.041***	− 0.027*	− 0.068***	0.105***
3 to 11 months	0.034	− 0.005	0.017	− 0.012	− 0.025	0.010	0.001	− 0.015
24 months and more	− 0.057***	0.008	− 0.011	− 0.021	0.000	0.003	− 0.019	0.014
Gender and youngest child in household (reference: woman w/o children in household)								
Mother with child aged 0 to 2 years	− 0.136***	− 0.067***	− 0.078**	− 0.210***	− 0.044**	− 0.081***	− 0.125***	0.288***
Mother with child aged 3 to 17 years	− 0.020	0.007	− 0.009	− 0.046*	0.005	− 0.009	− 0.012	0.021
Father with child aged 0 to 2 years	0.066*	− 0.022	0.081**	− 0.037	0.049*	0.031	0.019	− 0.018
Father with child aged 3 to 17 years	0.022	− 0.013	0.059*	− 0.012	0.037*	0.016	0.008	− 0.032
Man without children in household	0.015	− 0.010	0.048**	− 0.002	0.033**	0.023	0.010	− 0.014
Partner in household (reference: none)	− 0.057***	− 0.045***	− 0.044**	0.011	− 0.007	− 0.033**	− 0.030*	0.041**
Underemployment rate in federal state	− 0.010***	− 0.004*	− 0.015***	− 0.015***	− 0.004*	0.008***	− 0.004*	0.012***
Year (reference: 2015)								
2016	− 0.018	0.005	0.005	− 0.001	0.012	0.003	0.006	0.010
2017	− 0.020	0.002	0.009	− 0.013	0.018	0.008	0.001	0.025
2018	− 0.024	− 0.013	− 0.024	− 0.057**	0.006	0.000	0.008	0.027
2019	− 0.004	− 0.027*	− 0.023	− 0.050**	0.030*	0.011	0.019	0.029
2020	0.034	0.000	0.042*	− 0.017	0.050***	0.052***	0.072***	− 0.027
Pseudo-R^2^	0.032	0.019	0.029	0.040	0.034	0.034	0.064	0.041
N (person-waves)	8226	8224	8221	8207	8228	8200	8225	8234

Source: IAB, PASS, Welle 14 v1, 2015–2020. Own calculations

*p < 0.05; **p < 0.01; ***p < 0.001. Logit model, reported as average marginal effects. The significant coefficients can be read as the impact of a one-unit change of the independent variable on the estimated probability of receiving the offer by the Jobcenter

^a ^“No offer” means “financial support to become self-employed” and “other offers” have also not been granted, neither integration or language courses

With regard to ALMP programs, the pattern varies. Contrary to the descriptive results, the model with all control variables shows that non-recent immigrants are most likely to report *support with their applications*, while recent immigrants are least probable to do so (+ 5.7 p.p. respectively − 5.3 p.p. compared with native clients),^18^ in part falsifying H #3. In line with H #4, both recent and non-recent immigrants have a significantly lower likelihood of Jobcenters reimbursing application or travel costs than natives (− 20.1 p.p. and − 11.4 p.p.). Probably, native clients receive invitations for job interviews more often than immigrants (and then have the costs reimbursed), in spite of receiving fewer referrals and less support with applications from Jobcenters than non-recent immigrants.

Subsidized programs with employers or internships could help jobless immigrants gain employers’ trust, which they possibly do not enjoy as much as native applicants. However, multivariate regression yields no difference between the three client groups for this costly ALMP measure. Hence, despite the bivariate result that the recently immigrated reported this offer above average, H #5 is not confirmed when controlling for the other covariates in the model. But there is no inequity in access either.

Regarding activation or placement vouchers (for, e.g., occupational orientation, further vocational training), newly settled immigrants face lower chances (− 6.9 p.p.) of receiving respective offers compared with natives, despite their hypothesized higher need for such measures due to lacking knowledge about the local labor market and job culture or because of non-recognized foreign credentials. We can only speculate whether these offers are crowded out by other programs (e.g., integration courses might partly cover labor market topics) or whether caseworker consider recent immigrants’ knowledge of German insufficient for following programs offered in German. As longer-settled immigrants face those obstacles to a lesser extent than new arrivals, it appears plausible that they are not treated differently from natives. Yet, H #6-plus still needs to be dismissed.

Jobcenters offer the more extensive programs of “vocational (re-)training or a course” comparatively often to recent immigrants, with a likelihood 13.9 p.p. higher compared with natives and non-recent immigrants’ probability increased by 11.5 p.p., respectively. This finding chimes with H #7a, which supposes an especially high need and motivation on the part of immigrants (however, see chapter 4.3.1).

Finally, recent immigrants (but not the longer-settled) remain significantly more often (+ 10.1 p.p.) without any ALMP offer than natives in a given year of observation (integration and language classes excluded).

Table 2 reports the effects of control variables which are, however, of no central interest here. Let us just mention that most ALMP measures are more likely offered by Jobcenters to clients with a higher level of qualification rather than a low level. Jobcenters do not try to compensate the largest gaps in education by their measures, otherwise, those with only lower-secondary education would be more in their focus. Control variables contribute to the statistical explanation of the dependent variables’ variance,^19^ increasing the Pseudo-R^2^ compared to the gross models with migration experience as the only predictor (see Additional file 1: Table S1). Overall, when there already is a significant bivariate correlation, the effects of migration status on Jobcenter offers remain statistically significant with regard to most outcome variables when the full multivariate models consider the composition of the groups. The less frequent referrals to marginal employment of recently arrived immigrants (compared to natives) are explained by the composition of that group (e.g., the large share without professional qualification) to a larger extent than by migration-related factors. By contrast, support with applications does not differ between the two groups at first sight, but turns out statistically significant in the full model, such that recently-arrived immigrants receive fewer offers of this kind than natives, given their social composition. Overall, the explanatory power of our models with regard to Jobcenters’ offers is not very high. This hints at the importance of unobserved factors, probably linked to the dynamics of the service interaction, clients’ personal preferences, and case managers’ professional attitudes (Dietz and Osiander 2019) as well as the particular “cultures” of individual Jobcenters.^20^

One main finding of this section is that recent immigrants generally receive fewer offers from out of the ALMP toolbox than native clients. As for the longer-settled immigrants, the pattern of Jobcenter activity in some respects looks like an attempt to compensate the labor market obstacles that still confront this group, possibly because their linguistic and cultural knowledge is already higher than that of recently arrived immigrants, making non-migrant specific offers more feasible in the eye of the Jobcenter.

#### Comparisons among immigrants

In order to find out more about how Jobcenters react to the different profiles of immigrant clients, this section will explore the pattern of proposed ALMP measures *within* the immigrant subgroup of our sample. Restricting our estimations to immigrants allows us to extend our estimation models by some variables which are only meaningful for this group: legal status, duration of stay in Germany, and the self-perceived level of German language skills.^21^ As for the dependent variables, we now also look at offers reserved for immigrants, that is integration courses and other German language classes.

A long *duration of stay* has a significant impact: Referrals to regular employment or apprenticeships are more likely (+ 11.1 p.p.) if immigrants have lived more than 10 years (rather than less long) in the country (Table 3). For them, offers of minor employment also have a probability (7.0 p.p.) higher than that to more recently arrived individuals. By contrast, longer-settled immigrants face a higher risk of not reporting vocational (re-)training and other courses (− 13.2 p.p.), and, of course, receive significantly fewer offers to participate in integration or language courses. Interestingly, there is no significant difference between the most-recently arrived and the immigrants staying between 5 and 10 years, apart from the more frequent offer of integration or language courses to the former group. It is only due to these courses that the most recently immigrated have a lower probability (− 6.6 p.p.) than less recent arrivals of reporting no offer at all.

**Table 3:** Determinants of various ALMP measures by Jobcenters to job-seeking recipients of basic-income support (immigrants only)

Independent variables	Dependent variable: referral/offer made by the Jobcenter								
	Regular employment	Marginal employment	Assistance in applications	Reimbursement of application or travel costs	Program with employer or internship	Activation or placement voucher	Vocational (re-)training course	Integration or language course	No offer^a^
Legal status (reference: not EU citizen, fixed-term residence)									
German citizen	0.028	0.028	0.04	0.035	− 0.056*	0.032	− 0.036	− 0.154***	0.024
EU citizen, non-German	0.046	0.018	0.04	0.047	− 0.047*	0.046*	0.000	− 0.174***	0.036
Not EU citizen, open-ended residence	− 0.013	− 0.02	0.110*	0.000	− 0.082**	0.048	0.038	− 0.146**	0.042
Duration of stay in Germany so far (reference: 5 to below 10 years)									
0 to below 5 years	− 0.038	0.000	− 0.044	− 0.03	− 0.014	− 0.011	− 0.018	0.188***	− 0.066**
10 years and more	0.111**	0.070*	0.013	0.008	− 0.042	0.044	− 0.132***	− 0.302***	0.044
German language skills (reference: reasonable)									
Very good	0.061*	0.032	0.025	0.077*	− 0.018	0.013	− 0.031	− 0.127***	0.015
Good	0.037	− 0.005	0.039	0.076***	0.005	0.006	0.004	− 0.102***	0.017
Bad	− 0.051*	− 0.020	− 0.101***	− 0.100***	− 0.047*	− 0.042*	− 0.062*	− 0.022	0.036
Very bad	− 0.067	− 0.031	− 0.100*	− 0.172***	− 0.133***	− 0.028	− 0.130*	− 0.012	0.033
Age (reference: 35 to 44 years)									
18 to 24 years	− 0.016	0.019	0.013	− 0.008	0.049	0.027	0.013	0.027	− 0.008
25 to 34 years	0.000	0.005	− 0.004	− 0.01	0.003	0.009	− 0.018	− 0.021	0.020
45 to 54 years	− 0.014	0.01	− 0.024	− 0.031	− 0.004	− 0.039*	− 0.056*	− 0.034	0.025
55 to 64 years	− 0.104***	− 0.010	− 0.053	− 0.057	0.003	− 0.050*	− 0.123***	− 0.049	0.092***
State of health: bad (reference: very good to less good)	− 0.004	− 0.008	− 0.073	− 0.048	− 0.037	− 0.021	− 0.123**	− 0.049	0.015
Professional qualification (reference: none, lower-secondary school-leaving certificate at most)									
None, but upper or intermediate secondary school-leaving certificate	0.013	− 0.030	0.036	0.018	0.026	0.005	0.052*	− 0.048*	0.021
Non-academic professional qualification	0.056*	− 0.023	0.048	0.01	0.045*	0.023	0.057*	− 0.043	0.016
Academic qualification (university or technical/teacher training college)	0.008	− 0.060**	0.082***	0.077**	0.038*	0.035*	0.045	− 0.048*	0.002
Duration of current unemployment so far (reference: 12 to 23 months)									
0 to 2 months	− 0.001	− 0.002	− 0.024	− 0.001	− 0.028	− 0.008	− 0.048	− 0.128***	0.087***
3 to 11 months	0.054	0.009	0.043	0.008	− 0.021	0.006	− 0.003	− 0.048	0.026
24 months and more	0.014	0.025	0.027	0.018	0.008	0.011	0.004	− 0.006	0.025
Gender and youngest child in household (reference: woman w/o children in household)									
Mother with child aged 0 to 2 years	− 0.144***	− 0.057	− 0.071	− 0.123**	− 0.039	− 0.039	− 0.123*	− 0.099	0.187***
Mother with child aged 3 to 17 years	− 0.061	0.017	− 0.017	− 0.037	0.001	0.007	0.000	0.028	0.006
Father with child aged 0 to 2 years	0.035	0.004	0.124**	0.004	0.086**	0.050	0.034	− 0.005	− 0.034
Father with child aged 3 to 17 years	0.003	0.007	0.065	− 0.001	0.046	0.033	0.019	− 0.009	− 0.018
Man without children in household	0.023	0.012	0.055	0.016	0.061**	0.022	0.006	0.000	0.003
Partner in household (reference: none)	− 0.037	− 0.039*	− 0.056*	− 0.005	− 0.023	− 0.036*	− 0.070**	− 0.004	0.047**
Underemployment rate in federal state	− 0.003	− 0.002	− 0.009**	− 0.012***	− 0.005	0.006*	0.002	− 0.005	0.002
Year (reference: 2017)									
2015	0.046	− 0.024	− 0.030	− 0.052	− 0.021	− 0.009	0.075*	(base)	0.042
2016	0.030	− 0.008	− 0.023	0.012	− 0.007	0.007	0.063*	0.023	0.015
2018	0.018	− 0.031	− 0.006	− 0.048	− 0.024	0.014	0.048	0.044	− 0.014
2019	0.049	− 0.053**	− 0.047	− 0.056*	− 0.006	0.025	0.041	− 0.012	0.014
2020	0.143***	0.005	0.056	− 0.003	0.036	0.079***	0.119***	0.027	− 0.033
Pseudo-R^2^	0.049	0.034	0.037	0.034	0.047	0.036	0.043	0.247	0.104
N	3191	3187	3185	3176	3193	3172	3190	2886	3195

Source: IAB, PASS, Welle 14 v1, 2015–2020. Own calculations

*p < .05; **p < .01; ***p < .001. Logit model, reported as average marginal effects. The significant coefficients can be read as the impact of a one-unit change of the independent variable on the estimated probability of receiving the offer by the Jobcenter

^a^“No offer” means “financial support to become self-employed” and “other offers” have also not been granted

Appendix Table 7 shows that self-assessed German language skills differ widely between immigrant Jobcenter clients. Corroborating H #9, support with applications is less probable for persons with bad or very bad German skills (− 10 p.p. compared with clients with “reasonable” German skills). There is no significant limitation of referrals to minor jobs in case of clients with weak German language skills, but a reduced likelihood of reimbursing job-searching costs points to employers’ under-average response to those persons’ applications. In addition, clients with “bad” or “very bad” German skills are less likely (− 4.7 p.p. and − 13.3 p.p.) to report being offered programs with employers or internships than clients with “reasonable” German. As for vocational (re-)training and other courses, the probability of offers diminishes, too (− 6.2 p.p. and − 13.0 p.p.). It seems that caseworkers prioritize integration and language courses if clients hardly speak German, and contemplate ALMP options as soon as a certain level of German language has been reached. Possibly, Jobcenters consider the possession of some German skills as the condition of being able to participate in most ALMP programs. The results on integration and language courses also reflect the regulation that recent immigrants with good knowledge of German do not need to attend them. In addition, referrals to regular jobs are significantly less often made to immigrant clients with “bad” German skills (− 5.1 p.p.), but more often to those with “very good” ones (+ 6.1 p.p.). All in all, results on the impact of German language skills are consistent with H #9 regarding both ALMP measures and referrals to job vacancies.

*Legal status*, being a German citizen or not, having a permanent or fixed-term residence permit, does not seem to affect most Jobcenters’ ALMP offers, as soon as other immigrant-specific factors are controlled for. The possibility that the client might (have to) leave Germany in the near future does not seem to discourage caseworkers. Counter to H #10, German nationals, EU citizens and third-country nationals with a permanent residence status are offered a program with an employer or an internship less often than clients with a temporary residence permit. The latter, mostly stemming from Africa or the Middle East, are also offered an integration or language course significantly more often than all other clients.

All the remaining variables are identical to the ones used in Sect. 4.1, Table 2. We observe that the impact of clients’ qualifications differs from the full sample model (including natives) with regard to two measures: in the immigrant-only model, academics are not referred to regular employment more often than persons with very low education, and neither are academics significantly more likely of being offered activation or placement vouchers than less educated immigrants. Also, for other factors captured in variables, like bad health, age, or a partner in the household, we find fewer or weaker effects in the migrant-only model. Yet, this might be due to the smaller sample size.^22^

### Robustness tests

Given the limitations of the data already addressed in the methods section, we put our results to three tests of robustness. They include using a different regression model (4.3.2), a different sample (4.3.3), and an attempt to correct potential false responses to one survey item (4.3.1). Statistically speaking, our results presented above turn out to be trustworthy. In the case of the item “vocational (re-)training or a course”, a doubt remains in the case of immigrants in our PASS-sample.

#### Reassessing offers of vocational (re-)training and other courses in the light of integration and language classes

In chapter 3, we already addressed the issue of a possible overlap of the response category *vocational (re-)training or a course* and the migrant-specific *integration or language courses*. Due to the order of interview items, clients who were only offered an integration or language course are likely to say “yes” when they are first asked about offers of *Vocational (re-)training or a course*”. Potentially, these individuals then tick “yes” a second time when they are concretely asked about an *Integration or language course* later on. As a test of robustness, we therefore eliminate all person-waves from our sample in which both offers are reported and re-run our regression model on this reduced sample, which now consists of 7449 person-waves, among which 2414 are migrants (820 person-waves less than the full sample).^23^ The results dramatically change concerning Jobcenters’ offer of *Vocational (re-)training or a course* (Table 4): instead of prioritizing newly arrived immigrant clients by 13.9 p.p. as compared to native clients (Sect. 4.1), they are now at a disadvantage of − 10.4 p.p. As for non-recent immigrants, the coefficient turns insignificant, i.e., Jobcenters do not treat them differently form native clients.

**Table 4:** Robustness test (I): determinants of various ALMP measures by Jobcenters to job-seeking recipients of basic-income support (joint model), modified responses to item “vocational training or a course”

Independent variables	Dependent variable: vocational (re-)training or a course	
	All sample persons^a^	Only sample persons who did not also tick the offer of integration or language class^b^
Person category (reference: native basic income recipients looking for a job)		
Basic income recipients looking for a job who immigrated during the past 4 years	0.139***	− 0.104***
Basic income recipients looking for a job who immigrated at least 5 years ago	0.115***	0.017
…		

Source: IAB, PASS, Welle 14 v1, 2015–2020. Own calculations

***p < 0.001. Logit model, reported as average marginal effects. The significant coefficients can be read as the impact of a one-unit change of the independent variable on the estimated probability of receiving the offer by the Jobcenter

^a^Repetition of results from Table 2 for comparison.

^b^Same control variables as in Table 2 (coefficients not shown due to their similarity to those in Table 2)

But how convincing are these alternative results, given that a part of the observations which have been eliminated may really have been offered *both* types of courses, in which case ticking both survey items with a “yes” would be the adequate answer? There is no way of knowing the exact share of respondents who says yes to *vocational (re-)training or a course*” but in fact meant only an integration/language course. Yet, there are some reasons to expect a high number of immigrant clients who were only offered integration classes, regarding both timing and language preconditions. An integration class usually takes six to seven month on average (Goethe Institut 2020). Depending on when it starts and how often clients and case managers talk to each other, the chance of being offered a subsequent vocational (re-)training in the same reference year of the survey should be limited. Concerning language skills, integration courses lead to the language level A2 or B1 (Goethe Institut 2020), which is usually not sufficient to follow the complex teaching in vocational training attended by native speakers as well. Furthermore, official statistics do not imply an overrepresentation of immigrants (restricted to those with non-German citizenship) among participants of PES-financed further vocational training (see introduction; BA 2019c). A final reason to expect the share of correct “double-yes answers” to be small can be derived from the research of Kasrin et al. (2021, p. 4). They find that the number of refugee Jobcenter-clients who attended further vocational training was only close to half of those attending a program with an employer. As we know from our data that recently-immigrated PASS respondents mention the latter offer relatively rarely (16.3 percent), the share of those receiving vocational (re-)training offers should be much smaller than the 39.8 percent reported above in Table 1. These arguments speak in favor of our argumentation that among respondents reporting both (re-)training and language classes many were in fact only offered the latter, which will have a bearing on the conclusions drawn below.

#### Random-effects model

As mentioned above in the methods section, our empirical findings are derived from the variance of phenomena between observations, i.e., person-waves. Theoretically, it could also be possible that Jobcenters’ offers are triggered by changes that happen in the lives of clients. In this case, a model that takes the longitudinal dimension of the panel data into account would be more adequate. As a robustness test, we apply a random effects model, which builds on the variance both between and within persons. The results do not change compared to our main model: it is the same coefficients that turn out statistically significant, and they have the same sign (Additional file 1: Table S3). The between component seems be dominant compared with within component. This may also be due to the fact that only a minority of respondents is observed for more than 1 year.

#### Only one wave per person

On average, immigrant persons remain in our sample for a smaller number of waves. In order to check whether this could (partly) explain our results, we perform an extra regression analysis where each person is observed only once across the whole observation period, no matter if they satisfied the sampling criteria in 1, 2, or even more years of observation. We find that the results do not differ much from the full model, no matter whether we use the first or last wave in which the sample person is observed (Additional file 1: Table S4). Regarding the impact of migrations status as seen in Table 2, significant coefficients are widely reproduced. The only exception is that longer-settled immigrants get more referrals to regular jobs in their first year of observation, whereas there is no significant difference compared with natives in the person-wave model (Table 2). As for effect sizes, they are also very close in the alternative models, with the models using the first observation of each person yielding slightly bigger effect sizes than the models using the last observation, especially for the newly arrived immigrants. This could mean that the effect of migration background on Jobcenters’ offers is higher in the beginning of a job search. Overall, the findings of our robustness checks underscore that the results in Sect. 4.2 are not driven by a difference within persons over time, but differences between persons.

## Conclusion

Based on survey answers given by job-seeking recipients of basic income, our analysis of the PASS survey in year 2015 to 2020 sought to identify determinants of ALMP offers made by German Jobcenters to its clients. Our special attention was on possible differences between native and immigrant clients as well as on the effect of duration of stay among the latter. Our joint model for all job-seeking basic income recipients included native clients, recent immigrants (having arrived in Germany up to 4 years before the interview) and longer-settled immigrants (having stayed for 5 years or more). Whereas aggregate administrative data, treating non-German citizen as one single group, suggested no relevant immigrant-native gaps (see BA 2019c as cited in the introduction of our contribution), our distinction by time since migration proved crucial.

Our multivariate analyses showed that *recent* immigrants generally receive fewer referrals to job vacancies and fewer offers from out of the ALMP toolbox than natives in a given year. For example, the likelihood of a caseworker suggesting such immigrants to apply for an advertised regular job is 12.3 percentage points (p.p.) lower,^24^ and the likelihood of offering an activation or placement voucher is 6.9 p.p. lower than regarding native clients. These results chime with some older, less differentiated findings on a negative effect of foreign or non-EU citizenship on benefitting from PES-sponsored (re-)training or vouchers (Kruppe 2009, p. 14; Möller and Walwei 2009, pp. 305, 308–309; Osikominu 2005, p. 61) as well as with previous research indicating some degree of ethnic discrimination by German administration (Grohs et al. 2016; Hemker and Rink 2017). Our interpretation is that many caseworkers, and probably employers, often perceive newly-arrived immigrants as not yet ready for entering the labor market, even though they do not question their eagerness and even assert that some barriers to employment which burden native Jobcenter clients are absent in the case of refugees (Boockmann and Scheu 2019, p. 409). Still, Jobcenters primarily offer integration and language courses, which often constitute both a right and an obligation, to recent immigrants.

There is one category that recently-immigrated respondents report significantly more often (+ 13.9 p.p.) than native Jobcenter clients. In the questionnaire, it is called “vocational training, retraining or a course”. On the one hand, it might be convincing that recent immigrants received this kind of offer particularly often, as this would reflect the special needs of a group that often lacks qualifications and certificates relevant for and/or recognized on the German labor market (Knuth 2021, p. 56). Case managers explicitly highlight refugees’ need for further training (Boockmann and Scheu 2019, p. 408). On the other hand, most other measures were offered less often to recent immigrants, even support with applications (− 5.3 p.p. compared with natives), despite such clients being probably little familiar with the culturally specific way one applies for jobs in Germany. An alternative explanation of recent immigrants’ high propensity to tick “yes” on the survey item “vocational training, retraining or a course” could be its overlap, possibly caused by the sequence of the respective survey items, with “integration course or another German course”. This could erroneously make the occurrence of “vocational training, retraining or a course” appear as relatively frequent among recent immigrants.^25^ There are indeed good reasons to conclude that a high proportion of persons reporting both items were actually *only* offered integration or language courses (see Sect. 4.3.1). Our alternative model, excluding respondents with double positive answers, implies that Jobcenters are indeed likely to offer occupational (re-)training *less* often to recent immigrants than to natives or longer-settled immigrants, with no significant difference between the two latter groups. But certainly, future research should use better data, e.g., on the realization of Jobcenter-sponsored (re-)training, to verify this preliminary result.

Is there an alternative reading of our overall results with regard to the more recent immigrants? The low probability of referrals to open job positions to this group could be based on their preference not to work. We judge this as implausible because Jobcenters tend not to allow “laziness” on part of the clients, their main goal being to bring people into employment and to end benefit receipt. Moreover, migration research (e.g. Sinning 2011) and our own interviews with Jobcenter staff suggest that earning money and sending part of it back to family members in their home country is a major motivation and social obligation among immigrants. Similar to findings by Boockmann and Scheu (2019, p. 409), one of the interviewed experts put it this way:

*“Those who already gained certificates strive for having them recognized, but this is not their main focus. Their main focus is earning money.” (Interview II)*

Integration and language classes, i.e., the kinds of measures primarily proposed to recent immigrants, may be the first or second choice from the viewpoint of social law, Jobcenters, or immigrants themselves; they are often a necessary *interim* step to their successful labor market integration in the longer term.

With regard to longer-settled immigrants, Jobcenters seem to use the full breadth of their toolbox to bring their clients into employment. As regards assistance with their job search, immigrants who have been staying in Germany already for 5 years or more have a significantly higher probability of being referred to marginal (+ 6.0 p.p.) employment as well as being offered support with their applications (+ 5.7 p.p.), compared to native clients, with no statistically significant difference regarding open positions of regular employment. By contrast, they are less often (− 11.4 p.p.) offered a reimbursement of application or travel costs, which conveys that they are less often invited for job interviews by potential employers—an indicator of their disadvantages on the German labor market. The preferential support with applications offered to longer-settled migrants and the statistical similarity with native clients regarding most other ALMP measures suggests that there is no negative discrimination against these migrants by Jobcenters.

This reading is further backed by our finding that, as in accordance with the principle of equity, migrants’ world region of origin makes no significant difference for their probability of receiving ALMP offers, except for integration or language courses. The latter are significantly more often proposed to clients from Africa and Middle East/Asia, who overwhelmingly belong to the immigrant groups targeted by integration policy. Regarding ALMP measures which are not language-related, we do not find major differences by migrants’ legal status. The following statement of a caseworker interviewed for our project underlines this:

*Actually, as soon as the language part has more or less been dealt with, we treat immigrant clients just like the rest of our clients. We ask ourselves where the clients are heading, what would fit to them, what is reasonable, what are they able to achieve. (Interview II)*

This statement also points to the importance of sufficient knowledge of German as a facilitator of further ALMP measures, and in particular for chances of finding employment. Our deeper analysis of which immigrant-specific factors are linked to Jobcenters’ offers shows that referrals to regular employment and reimbursement of application expenses are indeed offered more often to immigrant clients the better their German skills are. Immigrant job-seekers with weaker German skills are also least likely to gain assistance in applications or programs with an employer or internships.

Overall, our analysis gives reason to believe that, compared with native clients, job-seeking immigrants receive overall equal treatment by the German PES, but only regarding longer-settled immigrants or those with (assumed or actual) good German language skills. Whether Jobcenters’ activities are actually sufficient and how closely they correspond to immigrant clients’ preferences deserves further investigation. At least in the eye of individual migrants willing to learn, up to 4 years since arrival without training-related support can feel like a long time and might cool off respective educational aspirations. In order for Jobcenters to institutionally measure up to recent immigrants’ high motivation to succeed in their host country, they should use an adequate mix of long-term oriented investment in human capital and short-term support to find work. ALMP measures combining employment, occupational qualification and advanced German language training seem promising (Boockmann and Scheu 2019, p. 418).

A desirable extension of our study would be to contrast ALMP measures offered with ALMP measures that are actually implemented. While our analysis of the former predominantly dealt with phenomena of selection, an extended analysis could analyze the determinants of self-selection that influence whether a client accepts an offer. Furthermore, we do not know when offers are used strategically by Jobcenters to merely test the availability of clients for the labor market. This would, of course, change the interpretation of our results as such activating ‘offers’ mean nothing positive from the clients’ viewpoint. However, we find it more plausible that Jobcenters’ offers are intended as supportive, especially regarding comparatively long and extensive ALMP programs.

Our analysis was restricted to years when refugees from Asia formed a large group among PES’ immigrant clients in Germany. It is up to future research to test whether our results hold once the structure of immigration changes again, e.g., if immigrants from European countries (like Ukraine) and family migrants from outside of Europe will again become the majority of recently arrived immigrants.

In addition, our time window analyzed is a historical boom phase of the German economy with low unemployment rates. Relatively few job seekers competed for the public resources dedicated to ALMP. If unemployment should rise again in the years to come, possibly as a consequence of geopolitical conflicts, ALMP spending per person will probably shrink even if aggregate expenditure grows (Lehwess-Litzmann 2018). No one can know at present whether the German labor market will deal with this as well as with the COVID-19 crisis.^26^ Jobcenters’ future immigrant clients will certainly need new occupational skills and certificates as well as employment experience in the German labor market.

### Supplementary Information

**Additional file 1: Table S1.** Gross model with migration experience as the only determinants of various ALMP measures by Jobcenters to job-seeking recipients of basic-income support (joint model). **Table S2.** Characteristics of immigrant basic-income recipients looking for a job, by combination of offers reported. **Table S3.** Robustness test (II): Random-effects mode (Odds ratios). Determinants of various ALMP measures by Jobcenters to job-seeking recipients of basic-income support (joint model). **Table S4.** Robustness test (III): Only one person-wave per person. Determinants of various ALMP measures by Jobcenters to job-seeking recipients of basic-income support (joint model).

## Data Availability

PASS data can be obtained from the IAB’s research data center under certain conditions, see https://fdz.iab.de/en/FDZ_Data_Access.aspx.

## Appendix

See Tables 5, 6 and 7.

**Table 5:** Basic-income recipients looking for a job: persons and person-waves in the sample by migration status

Person category	Number of persons	Number of times the person figures in the sample						Number of person-waves
		1	2	3	4	5	6	
Natives	2711	1496	610	301	146	110	48	5041
	100.0%	55.2%	22.5%	11.1%	5.4%	4.1%	1.8%	
Immigrants	2243	1.565	459	143	60	14	2	3234
	100.0%	69.8%	20.5%	6.4%	2.7%	0.6%	0.1%	
Total	4954	3061	1069	444	206	124	50	8275
	100.0%	61.8%	21.6%	9.0%	4.2%	2.5%	1.0%	

Source: IAB, PASS, Welle 14 v1, 2015–2020. Own calculations

**Table 6:** Basic-income recipient looking for a job: socio-demographic characteristics, by migration status (as % of the observed population)

Attributes	Natives	All immigrants	…Up to 4 years of stay	…At least 5 years of stay
Gender				
Male	52.5	59.8	73.4	46.7
Female	47.5	40.2	26.6	53.3
Age				
18–24	10.9	5.4	11.1	0.5
25–34	30.8	31.3	43.6	21.9
35–44	20.2	29.1	32.0	25.0
45–54	21.0	20.4	10.9	30.0
55–64	17.0	13.8	2.4	22.8
Professional qualification				
No professional qualification	39.4	54.9	61.2	47.8
… With lower-secondary school-leaving certificate at most	29.7	40.6	40.3	38.6
… With upper or intermediate secondary school-leaving certificate	9.7	14.3	20.9	9.2
Non-academic professional training	57.0	28.3	21.2	36.0
academic qualification	3.6	16.8	17.6	16.2
Self-reported (very) bad health	9.7	8.5	2.9	12.9
With partner in household	28.8	61.7	71.2	55.6
Children (by age) in household				
No children in household	62.3	38.8	37.6	40.1
At least one child aged 0–2	8.4	18.4	26.3	10.7
At least one child aged 3–17	29.4	42.7	36.1	49.1
Duration of current unemployment episode				
0–2 months	20.6	26.8	23.1	30.4
3–11 months	9.2	9.7	12.4	8.0
12–23 months	10.3	12.1	20.1	7.2
24 months and more	59.8	51.5	44.4	54.3
N (person-wave)	5041	3234	2202	1032

Source: IAB, PASS, Welle 14 v1. 2015–2020. Own calculations. The figures represent the mean over the weighted values for each year of observation

**Table 7:** Immigrant basic-income recipients looking for a job: legal status and countries of origin, by duration of stay (as % of the observed population of immigrants)

Attributes	All immigrants	…Up to 4 years of stay	…At least 5 years of stay
Legal status			
German citizen	13.2	3.0	21.8
Non-German EU citizen	19.4	18.6	22.4
Third country national with permanent residence permit	18.8	13.4	22.0
Third country national with temporary residence permit	48.6	65.0	33.7
Duration of stay (mean in years)	9.8	3.2	14.9
Region of origin			
Northern, Western and Southern Europe	3.2	3.1	3.8
Eastern Europe	20.1	16.7	26.0
Former Soviet Union	18.7	7.7	30.8
Other Asian country	45.0	62.5	24.8
Turkey	3.1	2.5	3.7
Africa	8.4	6.6	9.4
Other countries/unknown	1.5	1.4	1.6
German language skills (own account)			
Very good	12.2	7.7	15.4
Good	33.3	28.7	39.0
Satisfactory	38.6	46.6	30.8
Bad	13.4	13.5	12.3
Very bad	2.5	3.5	2.5
N (person-wave)	3234	2202	1032

Source: IAB, PASS, Welle 14 v1, 2015–2020. Own calculations. The figures represent the mean over the weighted values for each year of observation

## Abbreviations

ALMP: Active labor market policy
BA: German Federal Employment Agency (Bundesagentur für Arbeit)
EU: European Union
IAB: Institute for Employment Research
PASS: Panel Study Labour Market and Social Security
PES: Public employment services
SGB: Social Code (Sozialgesetzbuch)

## Footnotes

1 Statistics of the Federal Employment Agency (BA [*Bundesagentur für Arbeit*]) show that in the period from 2015 to 2019, 40.0 percent of new participants in ALMP attended measures for activation and professional integration (854,990 thousand individuals on the yearly average), among which, e.g., 5.8 percent participated in further vocational training and 10.3 percent in job-creation schemes. 34.1 percent received classic referrals to job openings (BA 2021, restricted to Sozialgesetzbuch [SGB] II, authors’ calculation).

2 Citizens from EU-countries make up 69.4 percent of the foreign population in 2020, with 42.8, 50.9, and 43.7 percent among those with a duration of stay of less than 1 year, between one and under 4 years, and between four and under eight, respectively. The equivalent percentages for Syrians are 7.2, 6.3, and 9.5 (see StaBa 2021, authors’ calculation).

3 Footnote on research project and funding institution as well as thanks to reviewers for their feedback.

4 Immigrants with the precarious status of asylum seeker and with extradition only temporarily suspended (*Duldung*) are the clientele of *Arbeitsagenturen* (SGB III) (see Boockmann and Scheu 2019, p. 404) and not considered here.

5 For Switzerland, Liechti et al. (2017, p. 264) show that foreigners profit no less from ALMP programs than natives regarding their ensuing job prospects.

6 Also, short-term measures can be strategically used by the PES to verify clients’ readiness to co-operate, the absence of which can lead to sanctions, which case workers are more inclined to apply to ethnic-minority clients (Linden 2021).

7 Non-EU family immigrants and asylum seekers recognized as refugees are currently the largest groups. Altogether, 238 thousand immigrants attended integration courses on the yearly average between 2015 and 2019 (BAMF 2020, and previous editions; authors’ calculation).

8 We do not know of any research on such cultural differences, but websites like that of Just Landed (2020) imply the respective need for information.

9 Citizens from EU-countries make up 69.4 percent of the foreign population in 2020, with 42.8, 50.9, and 43.7 percent among those with a duration of stay of less than 1 year, between 1 and under 4 years, and between four and under eight, respectively. The equivalent percentages for Syrians are 7.2, 6.3, and 9.5 (see StaBa 2021, authors’ calculation).

10 Immigrants having arrived as minors are left out because schooling abroad is a major contributor to migration-related problems on the labor market (Kalter and Granato 2018, on differences between first- and second-generation immigrants).

11 We do not test a hypothesis on the item of financial support to become self-employed, as the number of positive cases is too small for a multivariate analysis. For the same reason, we refrain from using the item “other offers”, which only 1.9 percent say to have received. Hence, the other categories in the PASS survey are almost exhaustive.

12 It remains unknown whether respondents also subsumed ‘1-€-jobs’ under this item, as the item battery does not explicitly mention this measure.

13 German language skills were coded as “very good” if respondents said that German was their first language; missing and implausible values were sorted into the medium category.

14 As the survey is offered in various languages, Turkish, Russian, and since wave 10 Arabic (Jesske and Schulz 2018, p. 74), its participation rate and validity regarding immigrants should be better than in German-only surveys.

15 Further correlations pertain to age and duration of unemployment (Person’s rho = 0.32) and a fairly but not prohibitively high correlation between having a partner and a combined variable which captures a persons’ sex and the fact of having children in the household (Cramer’s V = 0.55).

16 Beyond the sampling-induced clustering of person-years within persons, one could also control for treatment-induced clustering at the level of Jobcenters or case managers. However, such information is not provided in the data. If it could be included in the models, this might slightly change the estimated standard errors and significance of coefficients.

17 In the multivariate analyses, we sort the small groups of clients from Turkey to the Middle East/Asian category, while those from Northern, Western and Southern Europe are aggregated with Eastern Europeans.

18 This difference between these gross effects (see Additional file 1: Table S1) and net effects is partly due to the relatively high share of academics among recent arrivals, as high education makes reporting support for applications more likely, independently of migration status (see Table 2).

19 Other possible predictors like the partner’s employment status and household income turned out to have no influence.

20 There is no information on this in the PASS data. Matching the ADIAB extension to the PASS could provide us with the information on the responsible Jobcenter. We did not opt for this due to a loss of cases and a lack of administrative data in the case of recently-arrived migrants.

21 The information on world regions of origin would also be suited as a variable, but it is too highly correlated with the legal status to be simultaneously included. If regions are included instead of legal status, we do not find any effects of the former on ALMP offers, except that clients from Africa and Middle East/Asia are more often proposed integration or language courses. This, of course, should have to do with their legal status, which is omitted in these alternative models.

22 Also, the additional variables in the second model forbid a direct comparison of control variables’ impacts with the above model for the whole sample.

23 Additional file 1: Table S2 displays some differences of the characteristics of respondents, grouped by the combination of offers they reported: respondents who report both offers are more similar to respondents who only reported “integration or language course” in terms of legal status, duration of stay and German language skills, but more similar to respondents who only said yes to having been offered “vocational (re-)training or a course” in terms of professional qualification.

24 One should keep in mind that the survey item for referrals to open job positions also mentions dual vocational training. Future research should try to distinguish these options.

25 One can only speculate whether a confounding of language courses with vocational training might also be a reason why Kruppe (2009, p. 14) finds a small positive effect for recent immigration on the issue of training vouchers.

26 However, a particular consequence of the pandemic for migrants and refugees is revealed by Brücker et al. (2021, 26 and 30): These groups experienced a stronger rise of unemployment in the course of the year 2020, not primarily due to layoffs (net employment rates remained rather stable), but as a consequence of interrupted or cancelled integration, language and vocational training courses.
