# Supplementary material for: Jobcenters’ strategies to promoting the inclusion of immigrant and native job seekers: a comparative analysis based on PASS survey data
Source: J Labour Mark Res. 2022 Jul 11;56(1):9. doi: 10.1186/s12651-022-00313-8 (PMC9274638; doi:10.1186/s12651-022-00313-8)
Supplement: Supplementary file 1 — Additional file 1: Table S1. Gross model with migration experience as the only determinants of various ALMP measures by Jobcenters to job-seeking recipients of basic-income support (joint model). Table S2. Characteristics of immigrant basic-income recipients looking for a job, by combination of offers reported. Table S3. Robustness test (II): Random-effects mode (Odds ratios). Determinants of various ALMP measures by Jobcenters to job-seeking recipients of basic-income support (joint model). Table S4. Robustness test (III): Only one person-wave per person. Determinants of various ALMP measures by Jobcenters to job-seeking recipients of basic-income support (joint model). [file 12651_2022_313_MOESM1_ESM.docx]

# Additional file 1

Table S1: Gross model with migration experience as the only determinants of various ALMP measures by Jobcenters to job-seeking recipients of basic-income support (joint model)

| Independent variables | Dependent variable: referral/offer made by the Jobcenter | | | | | | | |
| --- | --- | --- | --- | --- | --- | --- | --- | --- |
|  | Regular employment | Marginal employment | Assistance in applications | Reimbursement of application or travel costs | Program with employer or internship | Activation or placement voucher | Vocational training course | No offer¹ |
| Person category (reference: native basic income recipients looking for a job) |  |  |  |  |  |  |  |  |
| Basic income recipients looking for a job who immigrated during the past 4 years | -0.103*** | -0.031*** | -0.005 | -0.171*** | 0.059*** | -0.055*** | 0.200*** | 0.046*** |
| Basic income recipients looking for a job who immigrated at least 5 years ago | 0.016 | 0.034* | 0.045* | -0.123*** | 0.006 | -0.022 | 0.107*** | 0.022 |
|  |  |  |  |  |  |  |  |  |
| Pseudo-R² | 0.009 | 0.004 | 0.001 | 0.018 | 0.007 | 0.006 | 0.036 | 0.002 |
| N | 8266 | 8264 | 8259 | 8247 | 8267 | 8240 | 8265 | 8274 |

Source: IAB, PASS, Welle 14 v1, 2015–2020. Own calculations. Explanatory notes: * p<.05; ** p<.01; *** p<.001. Logit model, reported as average marginal effects. The significant coefficients can be read as the impact of a one-unit change of the independent variable on the estimated probability of receiving the offer by the Jobcenter. ¹ “No offer” means “financial support to become self-employed” and “other offers” have also not been granted.

Table S2: Characteristics of immigrant basic-income recipients looking for a job, by combination of offers reported

| Selected characteristics | | offers reported by respondent | | | All immigrant clients |
| --- | --- | --- | --- | --- | --- |
|  |  | only integration / language course | only vocational (re-)training or a course | both |  |
| Duration of stay (years) | | 4.8 | 10.7 | 5.0 | 9.8 |
| German language skills (own account) (%) | Very good | 6.9 | 21.5 | 6.8 | 12.2 |
|  | Good | 23.6 | 33.4 | 29.7 | 33.3 |
|  | Satisfactory | 50.3 | 36.9 | 50.9 | 38.6 |
|  | Bad | 17.2 | 6.9 | 11.1 | 13.4 |
|  | Very bad | 2.0 | 6.6 | 1.9 | 2.5 |
| Legal Status (%) | German citizen | 6.5 | 15.6 | 7.4 | 13.2 |
|  | non-German EU citizen | 9.1 | 26.8 | 14.8 | 19.4 |
|  | third country national with permanent residence permit | 7.5 | 19.9 | 19.4 | 18.8 |
|  | third country national with temporary residence permit | 81.4 | 49.6 | 70.1 | 48.6 |
| Professional qualification (%) | no professional qualification | 64.6 | 42.6 | 52.0 | 54.9 |
|  | … with lower-secondary school-leaving certificate at most | 49.8 | 27.9 | 30.6 | 40.6 |
|  | … with upper or intermediate secondary school-leaving certificate | 14.8 | 14.7 | 21.4 | 14.3 |
|  | non-academic professional training | 18.1 | 28.9 | 31.1 | 28.3 |
|  | academic qualification | 17.3 | 28.6 | 17.0 | 16.8 |

Source: IAB, PASS, Welle 14 v1, 2015–2020. Own calculations; column percentage. Explanatory note: The figures represent the mean over the weighted values for each year of observation.

Table S3: Robustness test (II): Random-effects mode (Odds ratios). Determinants of various ALMP measures by Jobcenters to job-seeking recipients of basic-income support (joint model)

| Independent variables | Dependent variable: referral/offer made by the Jobcenter | | | | | | | |
| --- | --- | --- | --- | --- | --- | --- | --- | --- |
|  | Regular employment | Marginal employment | Assistance in applications | Reimbursement of application or travel costs | Program with employer or internship | Activation or placement voucher | Vocational training or course | No offer¹ |
| Person category (reference: native basic income recipients looking for a job) |  |  |  |  |  |  |  |  |
| Basic income recipients looking for a job who immigrated during the past 4 years | -0.782*** | -0.025 | -0.360*** | -1.244*** | 0.151 | -0.833*** | 0.938*** | 0.630*** |
| Basic income recipients looking for a job who immigrated at least 5 years ago | 0.217 | 0.495*** | 0.337** | -0.658*** | 0.135 | -0.134 | 0.838*** | -0.026 |
|  |  |  |  |  |  |  |  |  |
| Age (reference: 35 to 44 years) |  |  |  |  |  |  |  |  |
| 18 to 24 years | 0.191 | 0.476** | 0.604*** | 0.347* | 1.006*** | 0.072 | 0.068 | -0.710*** |
| 25 to 34 years | 0.099 | 0.206 | 0.216* | 0.340** | 0.274* | 0.071 | 0.100 | -0.342** |
| 45 to 54 years | -0.191 | 0.009 | -0.290** | -0.269* | -0.194 | -0.393** | -0.376*** | 0.420*** |
| 55 to 64 years | -0.548*** | 0.053 | -0.555*** | -0.515*** | -0.439** | -0.708*** | -0.925*** | 0.873*** |
|  |  |  |  |  |  |  |  |  |
| State of health: bad (reference: very good to less good) | -0.181 | -0.099 | -0.213 | -0.373** | -0.243 | -0.486** | -0.378** | 0.307* |
|  |  |  |  |  |  |  |  |  |
| Professional qualification (reference: none, lower-secondary school-leaving certificate at most) |  |  |  |  |  |  |  |  |
| None, but upper or intermediate secondary school-leaving certificate | 0.199 | -0.263 | 0.061 | 0.109 | -0.008 | 0.349* | 0.312** | -0.015 |
| Non-academic professional qualification | 0.402*** | -0.112 | 0.213* | 0.403*** | -0.011 | 0.361** | 0.160 | -0.219* |
| Academic qualification (university or technical / teacher training college) | 0.317* | -0.698*** | 0.554*** | 0.713*** | 0.061 | 0.845*** | 0.324** | -0.524*** |
|  |  |  |  |  |  |  |  |  |
| Duration of current unemployment so far (reference: 12 to 23 months) |  |  |  |  |  |  |  |  |
| 0 to 2 months | -0.324** | -0.235 | -0.358*** | -0.508*** | -0.455*** | -0.250 | -0.483*** | 0.650*** |
| 3 to 11 months | 0.272* | -0.082 | 0.122 | -0.037 | -0.287 | 0.143 | 0.005 | -0.129 |
| 24 months and more | -0.279** | 0.087 | -0.018 | -0.074 | -0.013 | 0.088 | -0.082 | 0.035 |
|  |  |  |  |  |  |  |  |  |
| Gender and youngest child in household (reference: woman w/o children in household) |  |  |  |  |  |  |  |  |
| Mother with child aged 0 to 2 years | -1.024*** | -0.748** | -0.592** | -1.446*** | -0.650* | -1.352*** | -1.098*** | 1.789*** |
| Mother with child aged 3 to 17 years | -0.134 | 0.031 | -0.077 | -0.265* | 0.037 | -0.153 | -0.093 | 0.153 |
| Father with child aged 0 to 2 years | 0.524** | -0.166 | 0.504** | -0.124 | 0.496* | 0.379 | 0.147 | -0.163 |
| Father with child aged 3 to 17 years | 0.191 | -0.131 | 0.372* | -0.080 | 0.371* | 0.188 | 0.057 | -0.217 |
| Man without children in household | 0.132 | -0.116 | 0.308** | 0.026 | 0.321** | 0.223 | 0.066 | -0.134 |
|  |  |  |  |  |  |  |  |  |
| Partner in household (reference: none) | -0.398*** | -0.428*** | -0.279** | 0.063 | -0.074 | -0.363** | -0.215* | 0.291** |
|  |  |  |  |  |  |  |  |  |
| Underemployment rate in federal state | -0.059*** | -0.047** | -0.094*** | -0.086*** | -0.042* | 0.091*** | -0.025 | 0.073*** |
|  |  |  |  |  |  |  |  |  |
| Year (reference: 2017) |  |  |  |  |  |  |  |  |
| 2015 | 0.154 | 0.009 | -0.061 | 0.110 | -0.205 | -0.117 | -0.017 | -0.207 |
| 2016 | 0.042 | 0.029 | -0.048 | 0.089 | -0.073 | -0.057 | 0.014 | -0.107 |
| 2018 | -0.029 | -0.164 | -0.238* | -0.291** | -0.125 | -0.092 | 0.047 | 0.029 |
| 2019 | 0.134 | -0.310* | -0.218* | -0.206 | 0.112 | 0.026 | 0.145 | 0.020 |
| 2020 | 0.341** | -0.005 | 0.147 | -0.047 | 0.280* | 0.418** | 0.444*** | -0.308* |
|  |  |  |  |  |  |  |  |  |
| Intercept | -0.432 | -1.556*** | -0.320 | 0.644** | -2.030*** | -3.176*** | -1.349*** | -2.246*** |
|  |  |  |  |  |  |  |  |  |
| N | 8226 | 8224 | 8221 | 8207 | 8228 | 8200 | 8225 | 8234 |

Source: IAB, PASS, Welle 14 v1, 2015–2020. Own calculations. Explanatory notes: * p<.05; ** p<.01; *** p<.001. Random-effects logit model, reported as odds ratios.

Table S4: Robustness test (III): Only one person-wave per person. Determinants of various ALMP measures by Jobcenters to job-seeking recipients of basic-income support (joint model)

| Independent variables | Dependent variable: referral/offer made by the Jobcenter | | | | | | | |
| --- | --- | --- | --- | --- | --- | --- | --- | --- |
|  | Regular employment | Marginal employment | Assistance in applications | Reimbursement of applic. or travel costs | Program with employer or internship | Activation or placement voucher | Vocational training course | No offer |
| *Model with all observations of each sample person¹* | | | | | | | | |
| Person category (reference: natives) |  |  |  |  |  |  |  |  |
| Immigrant, at most 4 years of stay in Germany | -0.123*** | -0.007 | -0.053*** | -0.201*** | 0.015 | -0.069*** | 0.139*** | 0.101*** |
| Immigrant, at least 5 years of stay in Germany | 0.039 | 0.060*** | 0.057** | -0.114*** | 0.012 | -0.013 | 0.115*** | 0.008 |
| N | 8226 | 8224 | 8221 | 8207 | 8228 | 8200 | 8225 | 8234 |
|  |  |  |  |  |  |  |  |  |
| *Model with only the first observation of each sample person* | | | | | | | | |
| Person category (reference: natives) |  |  |  |  |  |  |  |  |
| Immigrant, at most 4 years of stay in Germany | -0.123*** | -0.007 | -0.099*** | -0.193*** | 0.015 | -0.069*** | 0.145*** | 0.086*** |
| Immigrant, at least 5 years of stay in Germany | 0.048* | 0.042* | 0.021 | -0.121*** | 0.000 | -0.020 | 0.144*** | 0.000 |
| N | 4920 | 4917 | 4913 | 4907 | 4919 | 4897 | 4915 | 4923 |
|  |  |  |  |  |  |  |  |  |
| *Model with only the last observation of each sample person* | | | | | | | | |
| Person category (reference: natives) |  |  |  |  |  |  |  |  |
| Immigrant, at most 4 years of stay in Germany | -0.096*** | 0.006 | -0.048** | -0.180*** | 0.017 | -0.059*** | 0.148*** | 0.078*** |
| Immigrant, at least 5 years of stay in Germany | 0.025 | 0.035* | 0.057** | -0.103*** | 0.021 | -0.013 | 0.131*** | -0.026 |
| N | 4924 | 4921 | 4916 | 4907 | 4921 | 4905 | 4919 | 4926 |

Source: IAB, PASS, Welle 14 v1 2015–2020. Own calculations. Explanatory notes: * p<.05; ** p<.01; *** p<.001. Logit model, reported as average marginal effects. The significant coefficients can be read as the impact of a one-unit change of the independent variable on the estimated probability of receiving the offer by the Jobcenter. ¹ Repetition of results from Table 1 for comparison. ² Only part of the full model is shown in the table
